# The LEAF questionnaire is a good screening tool for the identification of the Female Athlete Triad/Relative Energy Deficiency in Sport among young football players

**DOI:** 10.7717/peerj.12118

**Published:** 2021-09-03

**Authors:** Edyta Łuszczki, Pawel Jagielski, Anna Bartosiewicz, Maciej Kuchciak, Katarzyna Dereń, Artur Stolarczyk, Paweł Pakosz, Lukasz Oleksy

**Affiliations:** 1 Institute of Health Sciences, Medical College of Rzeszów University, Rzeszów, Poland; 2 Department of Nutrition and Drug Research, Institute of Public Health, Faculty of Health Sciences, Jagiellonian University Medical College, Kraków, Poland; 3 Institute of Physical Culture Sciences, Medical College of Rzeszów University, Rzeszów, Poland; 4 Orthopaedic and Rehabilitation Department, Medical University of Warsaw, Warszawa, Poland; 5 Faculty of Physical Education and Physiotherapy, Opole University of Technology, Opole, Poland

**Keywords:** Bone mineral density, Energy intake, Female athlete triad, Female athletes, Relative energy deficiency in sport, Resting energy expenditure

## Abstract

**Background:**

It has been noticed that Female Athlete Triad (Fat) and Relative Energy Deficiency (Red-S) in Sport are characterized by the symptoms of impaired endocrine-metabolic function and bone health in female athletes. In addition, it may be evaluated with a qualitative tool, such as Low Energy Availability in Females questionnaire (LEAF-Q) and quantitative measurements: bone mineral density (BMD), resting energy expenditure (REE), body composition, 24-hour dietary recall.

**Methods:**

The aim of this study was to assess the prevalence of Triad and Red-S using the LEAF-Q in youth female football players. Additionally, the difference in the BMD, body composition, REE and energy intake (EI) were assessed between the Triad/Red-S risk and not at-risk groups.

**Results:**

Almost two thirds (64.7%) of participants are classified as being at-risk for the triad according to their LEAF-Q scores. There were no statistically significant differences (*p* > 0.05) between most of the values among children from the analyzed groups. There was a statistically significant difference (*p* < 0.001) between the EI values among girls from the two analyzed groups: at-risk (1,773.18 kcal ±  232.57) and not at-risk (2,054.00 kcal ±  191.39). Girls who did not meet the energy intake recommendations were 10.00 as likely to be in the Triad/Red-S risk group.

**Conclusion:**

Early identification of Fat/Red-S symptoms by screening tools such as the LEAF questionnaire is important in protecting young athletes from long-term damage due to the progression of the risk factors associated with the Fat/Red-S.

## Introduction

American College of Sports Medicine states energy availability (EA) by means of the amount of energy left over and available for proper organism functions after the energy used for exercise is subtracted from the calories from the meals. Low energy availability (LEA) in endurance athletes could be affected by eating disorders. This all together could have health consequences related to the Female Athlete Triad (FAT) (Loucks, 2004; Marcason, 2016).

The FAT is the condition regularly noticed in physically active girls and women and its is observed with 3 disorders: menstrual dysfunction, low energy availability and decreased bone mineral density (Nazem & Ackerman, 2012). The FAT is most common in girls in the sport disciplines, such as: ballet, swimming, gymnastics, long distance runners. Depending on the football position, players covers about 10–13 km during a match, (with high intensity running) (Burke, Loucks & Broad, 2006).

Potential symptoms of FAT contains: fatigue, disordered eating, dry skin, hair loss, rapid weight loss, orthostatic hypotension, anemia, amenorrhea (Logue et al., 2019). The main issue is the LEA. It decreases proper bone mineral density (BMD), could affect the hypothalamus’s output of gonadotropic hormones reducing the amount of estrogen, irregulating the menstrual cycle (Dusek, 2001). In the literature, authors noticed that girls who involve physical activity that emphasize leanness, the occurrence of secondary amenorrhea was 69%. In addition, women with low BMD and menstrual problems are uncovered to stress fractures more often (Rauh, Nichols & Barrack, 2010; Kraus et al., 2019).

The International Olympic Committee expert working group presented a new expression for the FAT named Relative Energy Deficiency in Sports (Red-S). The syndrome of Red-S may affect physiological functions, such as: menstrual cycle, metabolic rate, bone health, protein synthesis, immunity and cardiovascular risk. Red-S leads to a progressive disruption of performance (*e.g.*, by increased risk of injury, decreased coordination and concentration) (Mountjoy et al., 2014).

Evidence from the literature shows that FAT and Red-S affect an athlete at higher risk of injury and bone stress fractures and that’s why it is especially important to prevent early. Decrease in performance, weight loss, mood changes, common injury are the main symptoms, and should be noted during the physical examinations and at annual health checkups (Bachrach, 2007).

It was noticed that FAT and Red-S are characterized by the symptoms of impaired endocrine-metabolic function and bone health in physically active women. In addition, it could be assessed with qualitative tools, such as Low Energy Availability in Females questionnaire (LEAF-Q) and quantitative measurements: bone mineral density, resting energy expenditure (REE), body composition, 24-hour dietary recall (Heikura et al., 2018). The LEAF questionnaire is the most popular screening tool. It determines the risk of LEA by evaluating the symptoms connected with energy deficiency (*e.g.*, injury history) (Melin et al., 2014; Heikura et al., 2018). In addition, BMD examined by dual-energy X-ray absorptiometry (DXA) and REE level by indirect calorimetry led to provide suppressed metabolism (Ackland et al., 2012).

Previous studies highlights the necessity to detect the prevalence of LEA, particularly among female athletes without compare the BMD, body composition, REE and energy intake between the Triad/Red-S risk and not at-risk groups according to the LEAF-Q. Therefore, it is impossible to determine whether the LEAF-Q is a good screening tool for early detection of FAT and Red-S before significant changes in BMD, body composition, REE and energy intake occur. Further, data assessing the prevalence of FAT and Red-S using the LEAF-Q in youth female football players, are limited. Additionally, knowledge on how being in risk or not at-risk groups may influence BMD, body composition, REE and energy intake is important and may help guide screening for those at elevated risk for health concerns associated with FAT and Red-S.

As the FAT and Red-S are very common in athletes and to the authors’ knowledge, no research has yet assessed the prevalence of FAT and Red-S in youth female football players in Poland, the aim of this study was to assess the prevalence of FAT and Red-S using the LEAF-Q in youth female football players. Additionally, the difference in the BMD, body composition, REE and energy intake were assessed between the Triad/Red-S risk and not at-risk groups.

## Materials & Methods

### Participants

In the sports school the study was conducted (Rzeszów, southeastern Poland) The research included children (13–18 years) during the 2018/2019 school year. The inclusion criteria were: age 13–18 years, female, training football ≥ 2 years and training about 3 times/week and playing a match once a week, consent of the parent/guardian, no history of any chronic illness, not taking any hormonal therapy in the past 12 months, and not taking any medications that affect BMD.

All parents/guardians (*n* = 56) of girls from the school received the invitation message. 34 consents were received, 22 did not meet the inclusion criteria. Participants’ sports Physical activity was previously described (Łuszczki et al., 2020).

Football positions in the study group were as follows: goalkeepers (*n* = 4, 11.8%), strikers (*n* = 6, 17.6%), defenders (*n* = 14, 41.2%), midfielders (*n* = 10, 29.4%).

Verbal and written information about the study was directed to the study participants and then they gave their informed written consent to participate in the study.

### Assessments

#### Weight, height, and REE

The weight, height, and REE assessments of the study sample has been published elsewhere (Łuszczki et al., 2020).

The height was measured 3 times with an accuracy of 0.1 cm (stadiometer Seca 213). REE was examined by means of the indirect calorimetry method (Fitmate MED, Cosmed).

#### BMD and body composition

The GE Healthcare Lunar iDXA scanner (dual energy X-ray absorptiometry) was used. BMD and body composition (percent body fat, fat-free mass) were analyzed by a certified technician using DXA. Data were collected as previously described in Bartosiewicz et al. (2021).

For data analyses, low BMD was defined as a BMD Z score between −1 and −2 SDs (Nattiv et al., 2007) and −2 SDs or less below age-matched reference data at any bone site (International Society for Clinical Densitometry).

BMI was calculated as a ratio of weight to height (kg/m2). International Obesity Task Force (IOTF) criteria were used to categorize underweight, normal body weight, overweight and obesity in the study group (Cole et al., 2000).

#### Questionnaire data

The 25 item LEAF-Q questionnaire identifies athletes at-risk for LEA by utilizing subsets of gastrointestinal symptoms, injury frequency and menstrual dysfunction. The questionnaire was validated in endurance athletes (78% sensitivity/ 90% specificity). According to the LEAF-Q, EA, bone health and reproductive function could be properly classified (Melin et al., 2014). The LEAF-Q score ≥8 indicates that an individual is at-risk for low energy availability and female athlete triad.

Energy intake (EI) data was collected by a 24-hour food recall method from three days (Foster & Bradley, 2018). Food records were entered and processed with a food composition database. Energy of the diet was calculated using the DIETA 5.0 software (IZZ, Warsaw). Energy intake was compared to the Nutrition Standards for the Polish Population, consistent with gender and age in this group and calculated taking into account appropriate physical activity (Jarosz et al., 2020). On this basis, participants were classified as those who met the demand for energy standard and those whose energy consumption was below the recommendations.

#### Goldberg cut-off method

The conception of the Goldberg cut-off method was presented in detail by Goldberg et al. (1991).

The evaluation of this method has been described in detail (Goldberg et al., 1991). Specifically, the steps of the group-level evaluation.

For our group of girls, the PAL index for the “Moderate” physical activity category was 1.8. The average BMRest index for our group was 1.65. The calculated cut-off points were:

Lower cut - off for EIrep: BMRest = 1.66

Upper cut - off for EIrep: BMRest = 1.95.

According to Gibbs, Williams & De Souza (2013), we decided to not exclude under-reporters from the studied sample. Such an approach could introduce a source of unknown bias into the dataset and is not recommended (Black et al., 1991).

#### Statistical analysis

Descriptive statistics: number (N), percentage (%), mean (*x*), median (Me), and standard deviation (SD) were used. The Shapiro–Wilk test was performed to check the normality of the data. Because of normally distributed variables differences between players who were deemed to be at-risk (≥8) and those who were shown to be not at-risk (≤7) were checked using the Student’s *t*-test for independent samples. In addition, the odds ratio OR and 95% confidence intervals (CIs) were assessed. The OR was used to determine whether meeting the energy intake recommendations is a risk factor for the Triad/Red-S. The statistical analyses were performed using PS IMAGO PRO 6.0 (IBM SPSS STATISTICS 26). Statistical significance was set at *p* < 0.05.

### Ethics

This research project was carried out in accordance with the Helsinki Declaration. The study was approved by the institutional Bioethics Committee at the University of Rzeszów (Resolution No. 8/04/2019). Both the participants and the guardians gave their informed written consent to participate in the study.

## Results

### Study group characteristics

A total of 34 girls aged 13 to 18 years participated in the study. The mean age of the respondents was 15.41 ± 1.42 years. Table 1 shows the results for the descriptive statistics for individuals for the selected variables.

**Table 1:** Characteristics of the study group.

	Mean	SD	Me	Q 25	Q 75	Min	Max
Age [years]	15.41	1.42	15.00	14.00	17.00	13.00	18.00
Weight [kg]	55.89	5.50	55.11	51.70	59.37	46.16	71.05
Height [cm]	166.38	5.74	166.00	162.00	172.00	155.00	177.00
BMI [kg/m^2^]	20.16	1.88	20.05	18.30	22.20	17.20	23.20
REE [kcal]	1599.56	218.61	1654.50	1414.00	1779.00	1219.00	1913.00
EI [kcal]	1872.29	255.32	1848.00	1648.00	2140.00	1507.00	2308.00
BF [%]	27.07	3.61	27.70	23.80	29.60	20.60	33.40
FFM [kg]	40.72	4.22	40.05	37.70	43.40	32.50	53.20
BMD [g/cm2]	1.17	0.09	1.15	1.11	1.25	1.05	1.41
Z-score BMD	1.24	0.82	1.25	0.50	1.90	−0.20	3.50

**Notes.**

Me median SD standard deviation Min sample minimum Max sample maximum REE resting energy expenditure BMI body mass index BMD bone mineral density EI energy intake BF body fat FFM fat-free mass

#### The findings

In Table 2 the key LEAF-Q responses are presented. Many athletes reported injuries (85.3%), menstrual changes were common with increased exercise and gastrointestinal disturbances were less frequent than menstrual disturbances and injuries.

**Table 2:** Responses to key components of LEAF-Q as reported by participants.

LEAF questionnaire component	Frequency (n)	Percent (%)
1. Injury history Number of days lost from participation due to injury in past year:		
0	5	14.70
1–7	14	41.18
8–14	3	8.82
15–21	5	14.70
>22	7	20.60
2. Menstrual function Exercise-related menstrual changes:		
No changes	22	64.70
Bleed less	6	17.65
Increased bleeding	5	14.70
Bleed less days	1	2.95
Number of periods in the last year (if still menstruating)		
12 or more	9	27.27
9-11	4	12.13
6-8	11	33.33
3–5	9	27.27
Have your periods ever stopped for 3 consecutive months or longer (besides pregnancy)?		
No, never	23	69.70
Yes, it has happened before	8	24.24
Yes, that’s the situation now	2	6.06
Are your periods regular? (Every 28th to 34th day)		
Yes, most of the time	20	60.60
No, mostly not	13	39.40
3. Gastro intestinal function Do you feel gaseous or bloated in the abdomen, also when you do not have your period?		
Rarely or never	22	64.80
Yes, several times a day	4	11.76
Yes, once or twice a week or more seldom	8	23.53

#### LEAF-Q scores

Almost the two thirds (64.7%) of participants are classified as being at risk for the FAT/Red-S according to the LEAF-Q scores.

The values of individual parameters in the groups with risk and not at-risk are presented in Table 3. There were no statistically significant differences (*p* > 0.05) between most of the values among children from the analyzed groups. There were statistically significant differences (*p* <  0.001) between the EI values among girls from the two analyzed groups: at-risk (1,773.18 kcal ± 232.57) and not at-risk (2,054.00 kcal ± 191.39).

**Table 3:** The values of individual parameters in the groups with risk and not at-risk.

	**RISK GROUP (*n* = 22)**					**GROUP NOT AT-RISK (*n* = 12)**					*p*
	**Mean**	**SD**	**Me**	**Min**	**Max**	**Mean**	**SD**	**Me**	**Min**	**Max**	
Age [years]	15.41	1.56	15.00	13.00	18.00	15.42	1.16	15.00	14.00	18.00	0.988
Weight [kg]	55.69	5.59	54.71	48.17	71.05	56.26	5.57	55.88	46.16	62.52	0.779
Height [cm]	167.77	5.85	167.00	155.00	177.00	163.83	4.76	163.50	158.00	173.00	0.054
BMI [kg/m^2^]	19.76	1.77	19.55	17.20	23.20	20.89	1.93	20.80	17.70	23.20	0.095
REE [kcal]	1577.09	220.96	1622.00	1219.00	1913.00	1640.75	217.50	1671.00	1227.00	1878.00	0.426
EI [kcal]	1773.18	232.57	1750.00	1507.00	2308.00	2054.00	191.39	2142.50	1645.00	2251.00	**0.001**
BF [%]	26.93	3.60	27.20	20.60	33.40	27.33	3.78	28.05	21.40	32.30	0.764
FFM [kg]	40.68	4.49	39.60	32.50	53.20	40.81	3.85	40.35	35.90	46.70	0.829
BMD [g/cm2]	1.16	0.09	1.12	1.05	1.41	1.19	0.08	1.23	1.08	1.31	0.308
Z-score BMD	1.15	0.85	1.10	−0.20	3.50	1.39	0.75	1.65	0.40	2.60	0.417

**Notes.**

Me median SD standard deviation Min sample minimum Max sample maximum REE resting energy expenditure BMI body mass index BMD bone mineral density EI energy intake FFM fat-free mass BF body fat *p* *p*-value, indicate significant values (*p* <0.05)

When analyzing the body mass categories according to the IOTF criteria, only one girl (3%) was underweight, and the rest of participants were with normal body weight. In the case of BMD, it was also found that all persons had normal BMD scores.

In the study group, 76.5% of participants did not meet the standards for the energy recommendation. Girls, who did not meet the energy intake recommendations were 10.00 as likely to be in the Triad/Red-S risk group (Table 4).

**Table 4:** Odds ratio values for energy intake recommendations in the study group.

Energy intake	Risk group N (%)	Group not at -risk N (%)	OR	95% CI
Below the nutritional standards	20 (76.9)	6 (23.1)	10.00	1.58-63.09
Normal	2 (25.0)	6 (75.0)	1	

**Notes.**

OR, the odds ratio (with 95% confidence interval).

However, according to the Goldberg cut-off method 67.6% of girls were under-reporting of energy intake. No one person scored above the upper cut-off point (over-reporting) (Table 5). Therefore, this results may providing diet reports of poor-validity.

**Table 5:** Results of the Goldberg cut-off method in the study group.

	N	%
<Lower cut (<1.66)	23	67.6
Lower-Upper cut (1.66–1.95)	11	32.4
>Upper-cut (>1.95)	0	0.0
Total	34	100.0

## Discussion

In 2014, Melin et al. published the Low Energy Availability in Females Questionnaire (LEAF-Q). This is a validated screening tool used to detect the FAT/Red-S. The questionnaire contains questions connecting to menstrual and gastrointestinal function as well as injury history (Melin et al., 2014). To date a few studies have been published using the full version of the LEAF-Q. However, the 90% specificity and 78% sensitivity of this tool shows a lot of promise and is likely to be widely used as the initial screening tool to detect FAT/Red-S. We did not find any article where LEAF-Q in youth football players as a screening tool was used.

In our study, 64.7% of participants are classified as being at-risk for the triad according to their LEAF-Q scores. Folscher et al. presented that LEAF-Q scores classified 44.1% of participants at-risk for the FAT/Red-S (Folscher et al., 2015). Similar, Thomas et al. considered 31.1% of the female athletes at-risk of developing the Triad (Thomas, Gonzalez & Ghigiarelli, 2021). This shows that our result presented a higher prevalence of FAT/Red-S in youth football players. It should be noted that our group was younger (mean age 15.41 years) than most in the literature (Logue et al., 2018). There are only a few studies conducted in this discipline. There reason could be that football is currently a more popular sport among men.

However, our results did not show statistically significant differences (*p* > 0.05) between the most of the values among children from the analyzed groups. This may mean that significant differences between the groups of young girls have not yet emerged. Prevalence of low BMD among female athletes ranges from 0 to 15.4% (with *z*-score < −2). This rises to 39.8% when z-scores are defined as between −1.0 and −2.0 (Gibbs, Williams & De Souza, 2013). Although sports, such as football, can influence BMD, our findings highlight that poor bone health develops over a long period, and changes in BMD may not be detectable over a short timeframe and that previous exercise and dietary (D_3_ and calcium supplements) interventions may stimulate bone mineralization sufficiently to maintain BMD (Logue et al., 2018). It is therefore all the more important to educate children belonging to the risk group about this issue and to introduce preventive measures.

In our study, there was a significant difference in energy intake between the groups. Girls in the risk group ate significantly less calories than those in the non-risk group. The average caloric consumption in the risk group was 1,773.18 kcal. This result is similar to the Condo et al. who obtained 1,869.21 kcal in the study in female Australian rules football players (Condo et al., 2019). This can be the starting point for eating disorders and low energy availability (LEA), which is very common in athletes. LEAF-Q does not measure actual LEA behaviors—eating disorders—but instead, symptoms related to LEA. It commonly measures LEA risk in large cohorts. It was found that almost 40% active females in Ireland were at-risk of LEA (Logue et al., 2019). Our results show that girls, who did not meet the energy intake recommendations were 10.00 as likely to be in the Triad/Red-S risk group. This is consistent with other researchers. Kwiatkowska-Pamula et al. presented that 93% of female judokas engage in excessive body mass reduction consuming between 40–60% of their dietary needs (Kwiatkowska-Pamuła et al., 2017). The estimation of energy requirements in football players is difficult, as it can increase or decrease depending on age, daily activity levels or body composition (Mielgo-Ayuso et al., 2015). On the other hand, when analyzing the body mass categories according to the IOTF criteria, only one girl in our study (3%) was underweight, and the rest of participants were with normal body weight. The literature has presented that low energy availability has the potential impact on physiological function, menstrual function, BMD and energy balance (Kiens & Wright, 2011; Mielgo-Ayuso et al., 2015). In addition, energy-deficient athletes may have normal weight due to physiological adaptations. One of these is decreased resting energy expenditure, therefore athletes can have body mass stability yet energy deficient (De Souza et al., 2014)). In our study, there was no statistically significant difference in the REE level between two groups.

Due to the fact that girls have recently started their sports career, it should be emphasized that the visible changes in body weight may not have started yet. Therefore, a screening tool such as the LEAF questionnaire is very much needed at this stage to catch some changes in for example diet, before the visible failures begin (Mountjoy et al., 2015).

### Limitations and future research

Selection bias might have led to reduced generalizability as only one school was included in the study. Our next study should be expanded with more schools from different geographical areas. As this study was cross-sectional, temporality and causality issues should not be considered. The data is self-reported and therefore dependent on the understanding of the questions and honesty of completion. Under-reporting of EI could include both under-recording and under-eating. Previous research demonstrated that under-eating is a significant contributor to systematic bias.

In the future research, there is a need for monitoring over a longer period of time without intervention to check the effectiveness of the screening tool.

## Conclusions

According to the LEAF-Q, the prevalence of Fat/Red-S in young female football players is on very high level. In our group, there are no significant changes in the body composition, bone density and resting energy expenditure yet. However, we have noticed the significant changes in the energy intake between the groups. Early identification of Fat/Red-S symptoms by screening tools such as the LEAF questionnaire is important in protecting young athletes from long-term damage due to the progression of the risk factors associated with the Fat/Red-S. Additionally, the results are a benchmark for the institutions involved in this sport discipline. The results are important for informing medical staff and for comparison to other screening tools.

##  Supplemental Information

10.7717/peerj.12118/supp-1 Supplemental Information 1 Raw data

## Additional Information and Declarations

### Data Availability

The following information was supplied regarding data availability: The raw measurements are available in the Supplementary File.
